# Evaluation of specific absorption rate and heating in children exposed to a 7T MRI head coil

**DOI:** 10.1002/mrm.29283

**Published:** 2022-06-06

**Authors:** Shaihan J. Malik, Jeffrey W. Hand, David W. Carmichael, Joseph V. Hajnal

**Affiliations:** ^1^ Biomedical Engineering Department School of Biomedical Engineering and Imaging Sciences, King's College London, St. Thomas' Hospital London UK; ^2^ Center for the Developing Brain School of Biomedical Engineering and Imaging Sciences, King's College London, St. Thomas' Hospital London UK

**Keywords:** 7T MRI, electromagnetic simulation, pediatric imaging, RF safety, thermal simulation

## Abstract

**Purpose:**

To evaluate specific absorption rate (SAR) and temperature distributions resulting from pediatric exposure to a 7T head coil.

**Methods:**

Exposure from a 297‐MHz birdcage head transmit coil (CP mode single‐channel transmission) was simulated in several child models (ages 3–14, mass 13.9–50.4 kg) and one adult, using time‐domain electromagnetic and thermal solvers. Position variability, age‐related changes in dielectric properties, and differences in thermoregulation were also considered.

**Results:**

Age‐adjusted dielectric properties had little effect in this population. Head average SAR (hdSAR) was the limiting factor for all models centered in the coil. The value of hdSAR (normalized to net power) was found to decrease linearly with increasing mass (R^2^ = 0.86); no equivalent relationship for peak‐spatial 10*g* averaged SAR (psSAR_10g_) was identified. Relatively small (< 10%) variability was observed in hdSAR for position shifts of ±25 mm in each orthogonal direction when normalized to net power; accounting for $B_{1}^{+}$ efficiency can lead to much larger variability. Position sensitivity of psSAR_10g_ was greater, but in most cases hdSAR remained the limiting quantity.

For thermal simulations, if blood temperature is fixed (i.e., asserting good thermoregulation), maximum temperatures are compliant with International Electrotechnical Commission limits during 60‐min exposure at the SAR limit. Introducing variable blood temperature leads to core temperature changes proportional to whole‐body averaged SAR, exceeding guideline limits for all child models.

**Conclusions:**

Children experienced higher SAR than adults for the 297‐MHz head transmit coil examined in this work. Thermal simulations suggest that core temperature changes could occur in smaller subjects, although experimental data are needed for validation.

## 1 INTRODUCTION

Magnetic resonance imaging is an important technique for the study of normal and abnormal brain development in infants and children because it allows the measurement of brain structure and function noninvasively. Ultrahigh‐field 7T MRI provides an important opportunity to advance this research by enabling improvements in spatial resolution to resolve smaller brain structures.

However, at 7 T there are greater risks from RF‐induced tissue heating, and the exclusive use of local transmit coils presents challenges that must be assessed to ensure safe operation. In 2017 CE and Food and Drug Administration 510(k) certifications were issued regarding restricted clinical use of a specific 7T system for patients with mass > 30 kg.^1^ The Food and Drug Administration also advises that static fields at or below 8 T pose no significant risk to subjects older than 1 month.^2^ Recently RF safety associated with imaging neonates at 7 T has been discussed,^3^, ^4^, ^5^ but there are few reports of imaging of young children in systems > 3 T.^6^, ^7^

National and international standards and safety guidelines^8^, ^9^ manage the potential thermal hazard from RF energy deposition by limiting temperature increases and, as a surrogate, specific absorption rate (SAR). In addition to their smaller size, infants and children differ from adults in that the water content of their tissues is higher. Because tissue dielectric properties are in general based on data from adults, this may affect the dielectric and thermal properties appropriate for children.

The resulting temperature increases in children are affected by the immaturity of their physiological systems and morphological differences compared with adults as well as uncertainty in the thermal properties of their tissues. The main physiological difference between children and adults, which affects their thermoregulation, is a lower sweating mechanism caused by a lower sweating rate per gland.^10^ There is also evidence that children demonstrate greater cutaneous vasodilatation, and thus, skin blood flow relative to adults.^11^, ^12^ As a result, their ability for dry heat loss through increased perfusion is similar to or greater than that of adults. Although the ratio of total blood volume to body mass is higher in children than adults (for children > 1 year it is in the range of 75–80 ml kg^−1^ ^13^ compared with ∼70 ml kg^−1^ in the adult male^14^), total blood volume is smaller in absolute terms in children. Hence, there is greater potential for the mean blood temperature to increase during exposure to RF fields in smaller subjects.

In this safety study for ultrahigh‐field MRI involving children, simulations involving a generic 7 T (adult) head transmit coil and seven child voxel models from the Virtual Population^15^ representing the age range 3–14 years are reported. The results of these child simulations are compared with those obtained using the adult male voxel model Duke also from the Virtual Population. The effect of the water content typical of children of this age group on tissue dielectric properties and changes in $B_{1}^{+}$ and SAR compared with values obtained using adult dielectric properties are investigated. Models of thermally stable children (in the absence of RF) in a background temperature of 22°C representative of the MRI environment are presented before investigating temperature increases resulting from 60‐min exposure to RF at the maximum head average SAR of 3.2 W kg^−1^, as this was found to be the most limiting SAR constraint.

## 2 METHODS

### 2.1 Voxel models

Five child voxel models with mass < 30 kg (namely, Nina, Roberta, Thelonius, Dizzy, and Eartha [all v1.0]), two child models with mass > 30 kg (Billie and Louis [both v1.0]), and the adult male model (Duke v3.1) all from the Virtual Population^15^ were used in this study. Details are given in Table 1. Nina is a morphed version of Roberta. The Duke model was used for tuning and matching the transmit coil.

**TABLE 1:** Properties of models used in the simulations (from Gosselin et al^15^)

	Nina	Roberta	Thelonius	Dizzy	Eartha	Billie	Louis	Duke
Age (years)	3	5	6	8	8	11	14	34
Mass (kg)	13.9	17.8	18.6	25.3	29.9	35.4	50.4	70.2
Height (m)	0.92	1.09	1.16	1.37	1.36	1.46	1.68	1.77
Number of tissues	97	66	76	137	75	112	182	305

### 2.2 Transmit coil model

The coil model used in this work was a 16‐rung high‐pass birdcage head coil, 190 mm long and 305 mm in diameter, with a shield 205 mm long and 370 mm in diameter. The coil was driven in quadrature using two orthogonal ports (with fixed 90° phase difference) in one end‐ring (distal relative to the subject's body) and tuned by inserting capacitors across gaps in both end rings located between the rungs. To reduce the power radiated from the coil and shield, these structures were placed centrally within a larger cylindrical shell 1500 mm long and 650 mm in diameter representing the bore of the MR scanner. All conductors were assumed to be copper with a conductivity of 5.997 × 10^7^ S m^−1^. The same model was used in a recent study of neonatal exposure.^4^

### 2.3 Numerical simulations

Electromagnetic (EM) and thermal simulations were carried out using Sim4Life v5.2.2.1924 (Zurich MedTech, Zurich, Switzerland) on PCs with 2.3‐GHz Intel Xeon Gold 5118 processors and 64 GB RAM. Acceleware finite‐difference time‐domain solvers (Acceleware, Calgary, Alberta, Canada) on a Nvidia Titan RTX graphics card (1.77 GHz, 24 GB memory, 4608 CUDA cores, 576 tensor cores) (Nvidia, Santa Clara, CA, USA) were used for EM simulations. Uniaxial perfectly matched layers absorbing boundary conditions set to “medium” were used at the edges of the computation domain. Nonuniform gridding was used; more details on step sizes, sensitivity to gridding, and convergence are found in the Supporting Information. Wideband excitation was simulated using a Gaussian pulse with center frequency 297 MHz and bandwidth 100 MHz when Duke was positioned “brain‐centered” with the corpus callosum at the center of the coil. Harmonic simulations at 297 MHz with the same coil tuning (for the Duke adult model) were then carried out for the child cases (ie, the coil was not retuned for child models, replicating the expected coil tuning encountered in practice).

**FIGURE 1:**
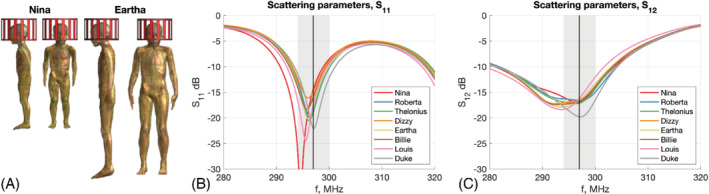
(A) Illustrations of Nina and Eartha child models positioned with brain centered within head coil. (B) Scattering parameter S_11_ for all models. (C) Scattering parameter S_12_ for all models. Note that S_21_ is identical to S_12_, and S_22_ was very similar to S_11_ in all cases, so they are not shown here. The coil was tuned and matched at 297 MHz (indicated by black line) using the adult model Duke. The resonance frequency shifts down when loaded with the child models; the light gray box indicates ±1% in frequency. The coil was not retuned for each model; – 297 MHz was used for all data presented

Each child model was positioned brain‐centered within the birdcage coil. Figure 1A shows the smallest (Nina) and largest (Eartha) child < 30 kg within the coil. Head regions used for computing head average SAR were defined manually by truncating at the neck; details of the volumes used for the child and adult models are listed in Supporting Information Table S2.

To investigate the sensitivity of SAR on the relative positions of the child models and birdcage coil, simulations were performed for the models shifted by ±25 mm in the left–right (LR), anterior–posterior (AP), and superior–inferior (SI) directions; additional ±50 mm SI shifts were also considered. As an example of the extreme SI shifts investigated, Supporting Information Figure S1 shows the Eartha model shifted by −50 mm and + 50 mm relative to the coil.

### 2.4 Dielectric properties

Although neonates have a much higher water content than adults, the difference decreases over the first 12–18 months of life such that water content of older children is much closer to adult values. Total body water (W_T_) values (in liters) can be predicted from age (A years), mass (M kg), and height (H cm)^16^:

(1) $$\ln\left(W_{T}\right)=C+0.551*\ln\left(M\right)+0.796*\ln\left(H\right)+0.008*A$$

where C is a constant value of −2.952 for males and −2.999 for females. Estimates of the dielectric properties of children^17^, ^18^ have been based on Lichtenecker's mixture formula^19^, ^20^ and a knowledge of W_T_, body mass, permittivity of water and adult tissues, and tissue density. In this approach, the relative permittivity of a child's tissue $\epsilon_{rCh}$ is found from

(2) $$\epsilon_{\mathrm{rCh}}=\epsilon_{\mathrm{rw}}^{\frac{\left(\alpha_{\mathrm{Ch}}-\alpha_{A}\right)}{\left(1-\alpha_{A}\right)}}\cdot \epsilon_{\mathrm{rA}}^{\frac{\left(1-\alpha_{\mathrm{Ch}}\right)}{\left(1-\alpha_{A}\right)}}$$

where $\epsilon_{\mathrm{rw}}$ and $\epsilon_{\mathrm{rA}}$ are the relative permittivities of water (74.3 at 37°C) and the corresponding adult tissue, respectively; and $\alpha =\rho W_{T}/M$ is the product of mass density with W_T_ per unit mass, taking value $\alpha_{\mathrm{Ch}}$ or $\alpha_{A}$ for child and adult, respectively.

Furthermore, the complex permittivity can be expressed as

(3) $${\hat{\epsilon }}_{\mathrm{rCh}}=\epsilon_{\mathrm{rCh}}^{\prime }-j\epsilon_{\mathrm{rCh}}^{\prime \prime }=\epsilon_{\mathrm{rCh}}^{\prime }-j\frac{\sigma }{2\pi f\epsilon_{0}}=\epsilon_{\mathrm{rCh}}^{\prime }\left(1-j\frac{1}{2\pi f\tau }\right)$$

where σ is the tissue conductivity at frequency *f*; $\epsilon_{0}$ is the permittivity of free space; and $\tau =\epsilon_{0}\epsilon_{r}/\sigma .$

Experimental data on permittivity and conductivity from a range of animal models of varying ages^21^, ^22^ suggest that τ for an individual tissue varies little with age. Because it is difficult to extrapolate from young animals to a human child's age, we have taken the mean value of τ over a range of tissue types and ages from Peyman's data (excluding data from newborn animals, which have relatively high water content), to arrive at the approximation $\tau_{\mathrm{Ch}}=0.94\tau_{A}$. Logically, τ_*Ch*_ for older teenage children will approach the adult value of 1; therefore, using this approximation in implementing Equation (3) for each tissue type in this work is biased to the younger child models.

In the case of the models studied here, the largest value of W_T_ occurs for Dizzy (see Supporting Information Table S3). Dielectric properties for this model were estimated using Equations (2) and (3) assuming that W_T_/M = 0.66 l kg^−1^ and 0.598 l kg^−1^ for Dizzy and Duke, respectively, and taking properties of adult tissues from the IT'IS database.^23^ In the case of tissues with low water content (ie, bone, red marrow, and fat), estimates of permittivity and conductivity were based on tissue water content. For example, the water content of cortical bone as a percentage of wet mass is about 15%–20% at age 5 years and gradually decreases by roughly one‐third over the next 10–15 years.^24^ In the case of fat, a 10% change water content between child and adult was assumed.

### 2.5 Thermal simulations

Thermal simulations were carried out for all child models and the adult model by solving the Pennes bioheat equation^25^ using the finite‐difference time‐domain solver within the *Sim4Life* software package.

The body responds to heating in a multitude of ways designed to maintain a constant core temperature; these mechanisms are collectively referred to as thermoregulation. Simulations via the bioheat equation are able to account for some of these mechanisms. Heat loss through sweating is not explicitly accounted for in the thermal solver within the *Sim4Life* package, but total heat loss to the environment is determined through a heat transfer coefficient (W m^−2^ K^−1^). The solver also provides options for temperature‐dependent perfusion and variable blood temperature. In this study, temperature‐dependent perfusion was implemented for skin and fat in all models using piecewise linear increases at 1° intervals and coefficients as described in Murbach et al,^26^ which are representative of an adult's vasodilation. This is a conservative approach to heat loss through vasodilation for children, as they demonstrate greater cutaneous vasodilatation, and thus, skin blood flow relative to adults.^11^, ^12^ For all other tissues, temperature‐independent perfusion rates were taken from the IT'IS database.^23^ Adult values for tissue specific thermal properties^23^ were assumed in the present study. This was also a conservative approach, because the thermal conductivity and heat capacity of a child's tissues would both be expected to increase with greater water content, leading to lower temperatures.

Thermal modeling via the bioheat equation for MRI applications typically uses a fixed blood temperature.^27^ The whole blood pool can be assumed to have a single temperature, as it circulates quickly throughout the body; holding this temperature constant implicitly assumes that the body maintains a constant core temperature by unspecified (and unmodeled) thermoregulation processes. To simulate a situation in which the core temperature can deviate, it is possible to incorporate a variable blood temperature into simulations, as demonstrated in previous studies.^28^, ^29^ Variable blood temperature can be enabled within the *Sim4Life* thermal solver via “use body core heating”; we refer to this option as “variable blood temperature” from here on. The calculation requires a defined total blood volume, which was computed using 75 ml kg^−1^ ^13^ for all child models (total blood volumes are listed in Supporting Information Table S4). To provide an independent measure of core temperature, in this work we computed this by averaging the temperature over the heart and brain.

Initial steady‐state temperature distributions for all thermal simulations were obtained by running for 60 min (from all tissue initial temperatures = 37°C) to steady state, assuming a background temperature of 22°C and a heat transfer coefficient of 8 W m^−2^ K^−1^. Temperature‐dependent perfusion and variable blood temperature options were inactive during these simulations, as we have found that these features prevent the simulation from reaching a reasonable steady state when activated from an unrealistic starting condition (4). The steady‐state temperature distributions obtained were then used as initial conditions for subsequent simulations.

Subsequent simulations were run for 60 min with options for temperature‐dependent perfusion in skin and fat, and variable blood temperature activated for the whole simulation time. The background temperature and heat transfer coefficient remained at 22°C and 8 W m^−2^ K^−1^, respectively.

Although there are reports of children becoming hypothermic during MR procedures, particularly if sedated,^30^, ^31^ a conservative approach was adopted in which the heat transfer coefficient between model and background (h = 8 W m^−2^ K^−1^) resulted in an essentially stable core temperature. For comparison with the adult case, simulations were performed using the Duke model under the same boundary conditions.

The power level during RF exposures was set to achieve the maximum SAR level for the normal operating mode according to the International Electrotechnical Commission (IEC) 60601–2‐33 standard.^9^ As outlined in the Results section, the most constraining SAR limit for all centrally located models (including the adult) was the head average SAR = 3.2 W kg^−1^. To achieve this, thermal simulations were run at constant power levels ranging from 15.2 W to 19.4 W for the child models and 21.3 W for the adult.

## 3 RESULTS

### 3.1 Electromagnetic simulations

Table 2 compares adult dielectric properties of tissues within the head region with those calculated using Equations (2) and (3) and adjusted for the total body water for the Dizzy model (using Equation [1]). The increases in both permittivity and conductivity for bone marrow, white matter, cartilage, eye lens, and skin ranged from about 8%–15%. For tissues with low water content (bone, fat, mandible, and skull), the increases were about 25%, and for the remaining tissues they were 1–4%.

**TABLE 2:** Comparison for Dizzy model of dielectric properties at 297 MHz for head tissues (assuming total body water per unit mass = 0.66  l kg^−1^) and adult values taken from Hasgall et al^23^

	Permittivity			Conductivity (S m^−1^)		
Tissue	Dizzy	Adult	Percent difference	Dizzy	Adult	Percent difference
Bone (cortical)^a^	16.8	13.5	24	0.103	0.082	26
Bone marrow (red)^a^	13.6	12.1	12	0.195	0.17	15
Brain (gray matter)	62.3	60.1	4	0.72	0.69	4
Brain (white matter)	48.0	43.8	10	0.45	0.41	10
Cartilage	51.3	46.8	10	0.60	0.55	9
Cerebellum	62.1	59.9	4	1.01	0.97	4
Connective tissue	51.6	48.0	8	0.58	0.54	7
Eye (lens)	43.4	38.4	13	0.40	0.35	14
Eye (vitreous humor)	69.8	69.0	1	1.53	1.52	1
Fat^b^	14.7	11.75	25	0.096	0.076	26
Hippocampus	62.3	60.1	4	0.72	0.69	4
Hypothalamus	62.3	60.1	4	0.72	0.69	4
Mandible^a^	16.8	13.5	24	0.103	0.082	26
Medulla oblongata	62.1	59.9	4	1.01	0.97	4
Midbrain	62.1	59.9	4	1.01	0.97	4
Muscle	61.0	58.2	5	0.81	0.77	5
Pineal body	64.4	62.5	3	0.88	0.85	4
Pons	62.1	59.9	4	1.01	0.97	4
Skin	54.1	49.9	8	0.69	0.64	8
Skull cortical^a^	16.8	13.5	24	0.103	0.082	26
Thalamus	62.3	60.1	4	0.72	0.69	4
Tongue	61.6	59.0	4	0.78	0.74	5

a Assuming 20% water for child and 15% water for adult.^24^

b Assuming a 10% increase in water content.

Table 3 compares the power budgets and SARs resulting from simulations of Dizzy, assuming either adult dielectric properties or the adjusted values listed in Table 2. Small changes in the power budget occurred (0.6% in absorbed power and 1%–2% in reflected/coupled power and radiated power) when the adjusted dielectric properties were used. The mean $B_{1}^{+}$ changed by 0.2%, and both head average SAR (hdSAR) and peak‐spatial 10*g* averaged SAR (psSAR_10g_) were reduced (by 1.1%–1.4% and 3.5%–3.7%, respectively). In light of the small effect on power budget details and SARs, adult dielectric properties were assumed in all of the simulations described subsequently.

**TABLE 3:** Comparison for Dizzy of power budgets, mean $B_{1}^{+}$, and SAR values assuming adjusted and adult dielectric properties for head tissues from Table 2

		Dizzy		
		Adjusted ϵ, σ	Adult ϵ, σ	Percent change
Power budget (%)	Absorbed	79.0	79.5	−0.63
	Reflected/coupled	4.7	4.5	1.04
	Radiated	11.4	11.2	1.8
	Other losses	4.9	4.8	2.08
Mean $B_{1}^{+}$ for 1 W total power (μT/√W)		0.486	0.485	0.21
SAR per W total power (W/kg/W)	hdSAR	0.178	0.180	−1.1
	psSAR_10g_	0.472	0.489	−3.5
SAR per mean $B_{1}^{+}$ (W/kg/μT^2^)	hdSAR	0.754	0.765	−1.4
	psSAR_10g_	2.001	2.078	−3.7

Table 4 summarizes the results of EM simulations for each model. The child models changed the loading, leading to a shift in the resonance frequency, as illustrated in Figure 1B,C, which show the S‐parameters for each model. The shifts are all within 1% and smaller than observed when loading the same coil model with a neonate^4^; others have reported birdcage coils with similar load sensitivity.^32^, ^33^ The proportion of power absorbed by the children was less than the adult, and there was a trend for increased absorption from the smallest to the largest child, correlated with a corresponding decrease in radiated power. Reflections, coupling, and other losses, such as ohmic losses in the coil, accounted for the remaining power.

**TABLE 4:** Summary for all models of power budgets, mean $B_{1}^{+}$, and SARs. Because the ratio of SAR limits psSAR10g/hdSAR = 3.125, a ratio < 3.125 implies that the hdSAR is the limiting parameter, which is the case for all models.

		Nina	Roberta	Thelonius	Dizzy	Eartha	Billie	Louis	Duke
Power budget (%)	Absorbed	72.8	77.6	78.6	79.5	82.3	81.1	83.9	87.9
	Reflected/coupled	4.6	2.4	3.0	4.5	3.9	4.8	3.8	0.4
	Radiated	18.2	15.1	14.4	11.2	10.3	10.6	8.2	7.0
	Other	4.4	4.9	4.0	4.8	3.5	3.5	4.1	4.7
Mean $B_{1}^{+}$ for 1 W total power (μT/√W)		0.535	0.505	0.495	0.485	0.499	0.506	0.501	0.477
$B_{1}^{+}$ coefficient of variation		0.17	0.24	0.25	0.17	0.20	0.18	0.20	0.19
SAR per W total power (W/kg/W)	hdSAR	0.211	0.207	0.199	0.180	0.172	0.180	0.165	0.150
	psSAR_10g_	0.517	0.526	0.446	0.489	0.474	0.472	0.515	0.448
	wbSAR	0.053	0.044	0.040	0.030	0.027	0.023	0.017	0.013
SAR per mean $B_{1}^{+}$ (W/kg/μT^2^)	hdSAR	0.736	0.811	0.813	0.765	0.692	0.703	0.657	0.659
	psSAR_10g_	1.806	2.059	1.821	2.078	1.907	1.851	2.052	1.972
	wbSAR	0.185	0.173	0.163	0.129	0.108	0.090	0.068	0.057
Ratio psSAR_10g_/hdSAR		2.5	2.5	2.2	2.7	2.8	2.6	3.1	3.0
Total power (W) required for hdSAR = 3.2 W/kg		15.2	15.5	16.1	17.8	18.6	17.8	19.4	21.3

Abbreviations: wbSAR, whole body averaged SAR; hdSAR, head averaged SAR; psSAR_10g_, peak spatial 10g averaged SAR.

For the children, the SAR per watt of total input power was similar to or greater than that for the adult, with hdSAR 10%–40% greater and psSAR_10g_ 5%–17% greater than the adult case. Whole body average SAR (wbSAR) was low for all models, as this is a local head transmitter, but increased as the subject mass decreased, commensurate with the larger fraction of the body exposed.

**FIGURE 2:**
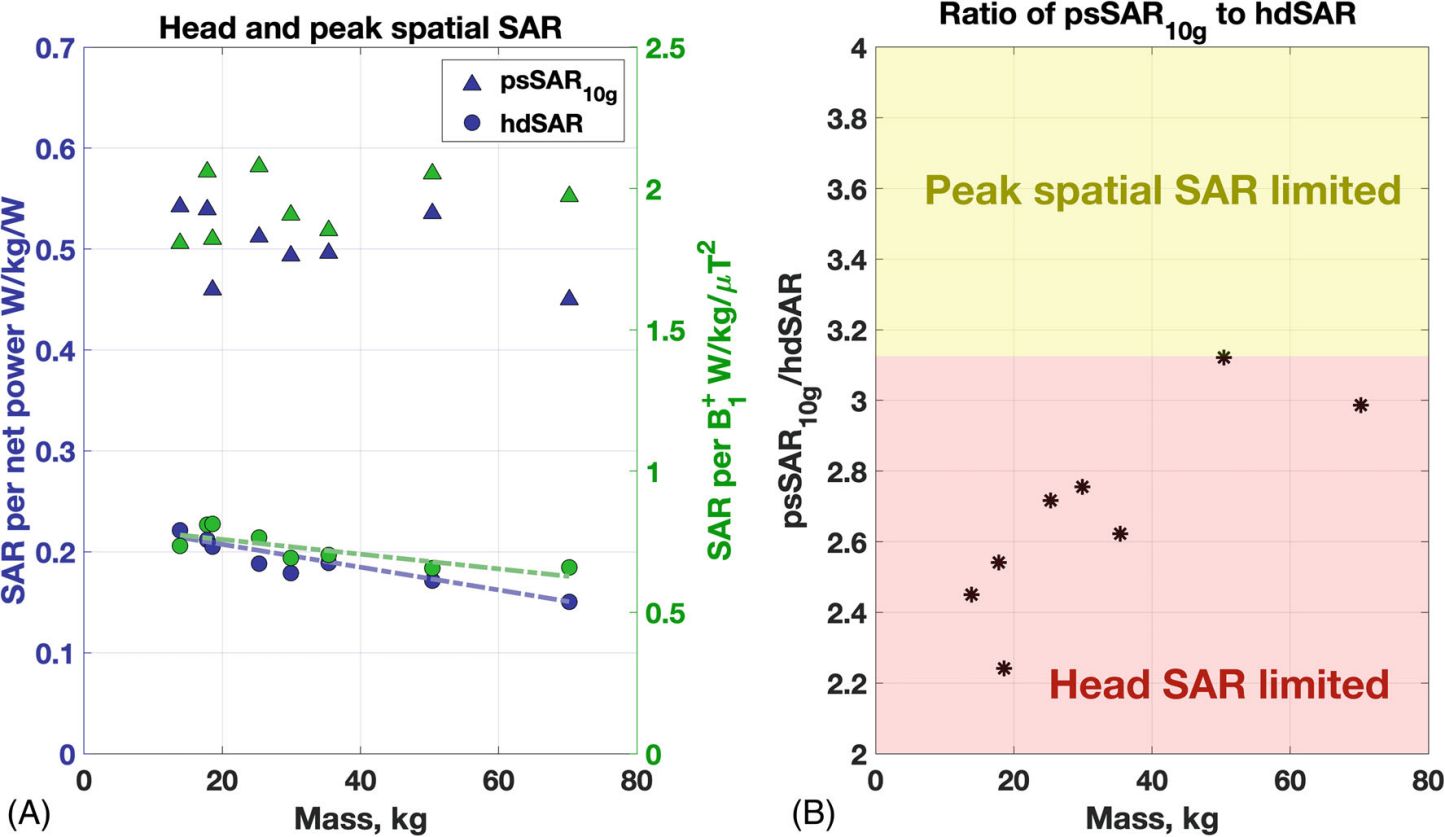
(A) Head average specific absorption rate (hdSAR) and peak spatial SAR (psSAR_10g_) as a function of model mass. Two normalizations commonly used for SAR estimation on commercial scanners are plotted ‐ to net power (ie, forward‐reflected) and to average $B_{1}^{+}$. Linear best fits for hdSAR versus mass are also plotted (dashed lines; see Equations [4] and [5]). (B) Ratio of psSAR_10g_ to hdSAR for each model. A ratio below 3.125 indicates that the limiting value is the hdSAR (International Electrotechnical Commission [IEC] limit 3.2 W kg^−1^) as opposed to psSAR_10g_  (IEC normal mode limit 10 W kg^−1^); all models are in this regime

There are a range of possible approaches for using results of this type of simulation for SAR prediction. One approach is to consider the SAR per unit net forward power (ie, accounting for reflections), and another is to normalize to the achieved $B_{1}^{+}$_._ Figure 2A summarizes the simulated SAR results graphically, using both of these normalization approaches. It can be seen that hdSAR is more strongly correlated with mass than psSAR_10g_, especially when normalized to net power rather than $B_{1}^{+}$. The following relationships could be obtained by fitting linear models to Figure 2A:

Net power normalized:

(4) $$\text{hdSAR}=0.23-1.13\times 10^{-3}\;M\;\left(R^{2}=0.86\right)$$

$B_{1}^{+}$ normalized

(5) $$\text{hdSAR}=0.81-2.60\times 10^{-3}\;M\;\left(R^{2}=0.58\right)$$

where *M* is the mass in kilograms. No strong correlation with mass for psSAR_10g_ was found.

Because the ratio of limits on psSAR_10g_ and hdSAR specified by the IEC^9^ is 3.125 (normal mode), the ratio of SAR values determines which will be the active limit in practice (noting that wbSAR is never the limit for this head transmit coil). As shown by Figure 2B, all models (including Duke) are limited by hdSAR, although for Louis the psSAR_10g_ and hdSAR are similar relative to their respective limits. Looking across models, there is a trend for the hdSAR to provide a more restrictive limit compared with psSAR_10g_ as subject mass reduces.

**FIGURE 3:**
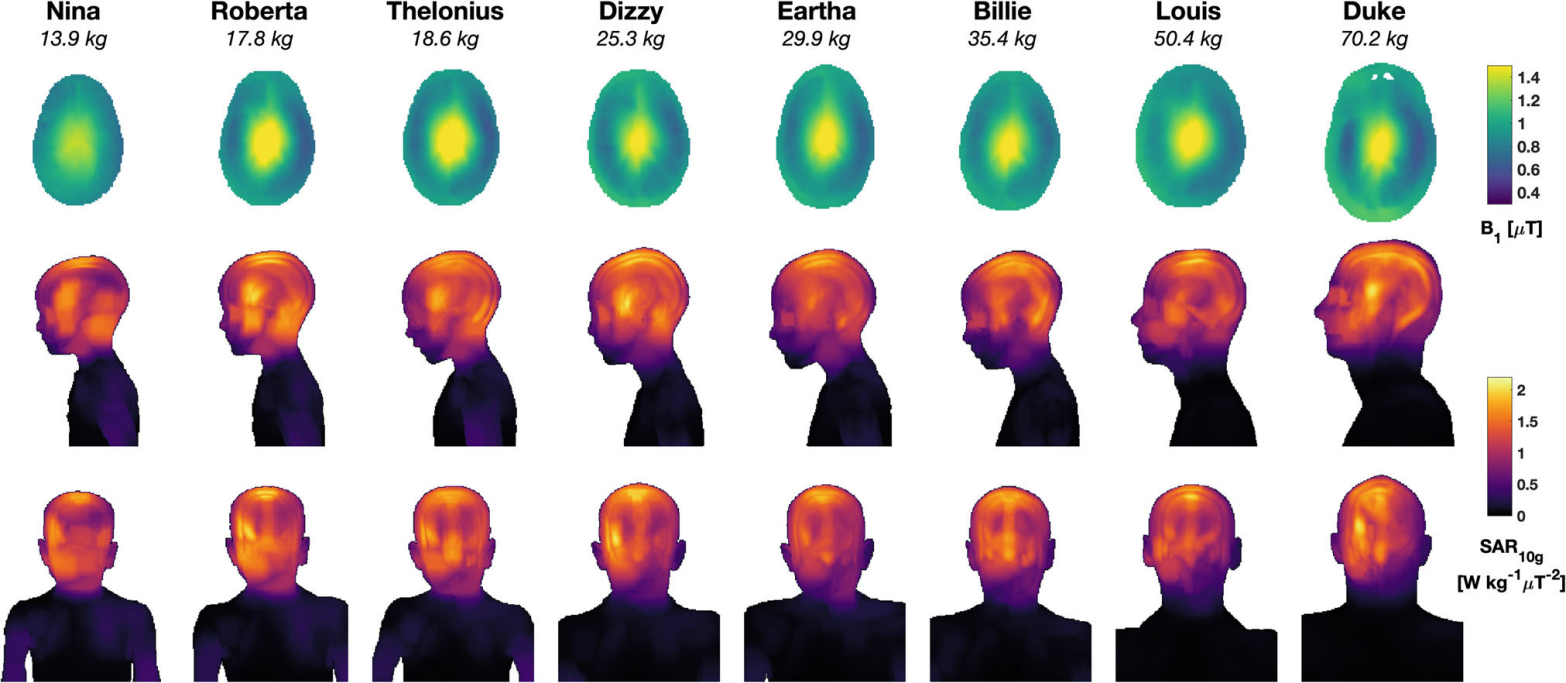
The $B_{1}^{+}$ and 10*g* averaged SAR (SAR_10g_) for all models, depicted at the same spatial scale. Top row: $B_{1}^{+}$ distributions in central axial plane for mean $B_{1}^{+}$ = 1 μT; this slice is the one used to normalize the SAR distributions also reported in Table 4. Upper row: $B_{1}^{+}$ in central transverse section; middle/bottom rows: SAR_10g_ distributions as maximum projections in sagittal/coronal views, respectively

Figure 3 shows the spatial distributions of $B_{1}^{+}$ within the central axial slice and projections of SAR_10g_. The inhomogeneity of $B_{1}^{+}$ for the child models was similar to that of the adult model, as quantified by the coefficient of variation in Table 4. In all cases, the psSAR_10g_ occurred in gray matter; a similar value for psSAR_10g_ occurred in CSF in the Roberta, Thelonius, and Duke simulations.

**FIGURE 4:**
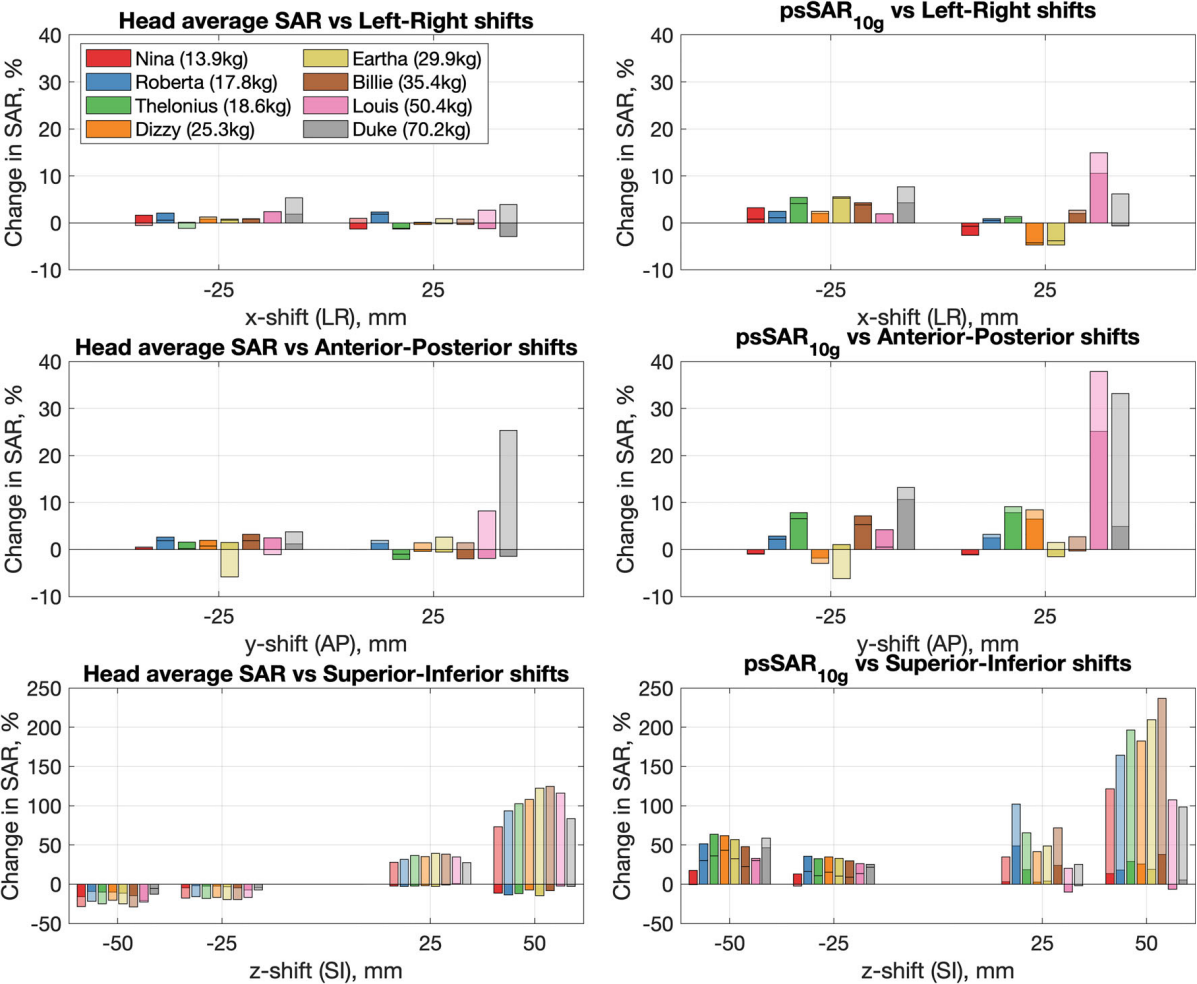
Changes in simulated SAR for all models shifted inside the coil. In each case, results are given for both the SAR normalized to net power and to $B_{1}^{+}$ (the latter are shown with transparent bars). In this case the $B_{1}^{+}$ was measured in the same anatomical slice (ie, it shifted with the subject). Typically the change in SAR normalized to $B_{1}^{+}$ was greater than that normalized to net power, if no transparent bar is visible that indicates the opposite was true. Note that the y‐axis scales for the bottom row are different than the other plots, as superior–inferior (SI) shifts lead to much larger changes. Abbreviations: AP, anterior–posterior; LR, left–right

Figure 4 summarizes the results for shifting the child model position within the head coil; the complete data set is tabulated in Supporting Information Tables S5.1–8. The figure presents data normalized both to net power (solid bars) and $B_{1}^{+}$ (transparent bars); in the latter case, the same anatomical slice was used for determining $B_{1}^{+}$ to simulate imaging of the same anatomy with an altered position of the subject. The smaller child models (mass ≤ 30 kg) had < 10% change in SAR regardless of normalization method for shifts of ±25 mm in the LR or AP directions, and for ±25 mm shifts in the SI direction when normalizing to net power. Normalizing for $B_{1}^{+}$ generally results in much larger positional sensitivity, particularly for SI shifts, because the $B_{1}^{+}$ efficiency of the coil is also adversely affected by shifting the subject. The larger child and adult models were generally more sensitive to position, particularly AP shifts, in which the face could get closer to the coil structure. Supporting Information Figure S2 illustrates the ratios of psSAR_10g_ to hdSAR for the shifted models. This shows that the hdSAR remains the limiting case for most models with AP and LR shifts; however, when shifted in the SI direction, psSAR_10g_ can become much larger, making the local SAR the limiting factor.

### 3.2 Thermal simulations

Initial simulations were performed to obtain a steady‐state temperature distribution in the absence of RF heating; a background temperature of 22°C and heat transfer coefficient 8 W m^−1^ K^−1^ resulted in a core temperature in the range of 37.2°C–37.3°C and mean skin temperature in the range of 34.3°C–34.4°C, conditions similar to reports in the literature.^12^ The core and maximum temperatures were essentially stable in the absence of RF (changes < −0.03°C over 60 min). Simulations were then performed using maximum RF exposure at the IEC guideline limit: hdSAR = 3.2 W kg^−1^ for all models.

**FIGURE 5:**
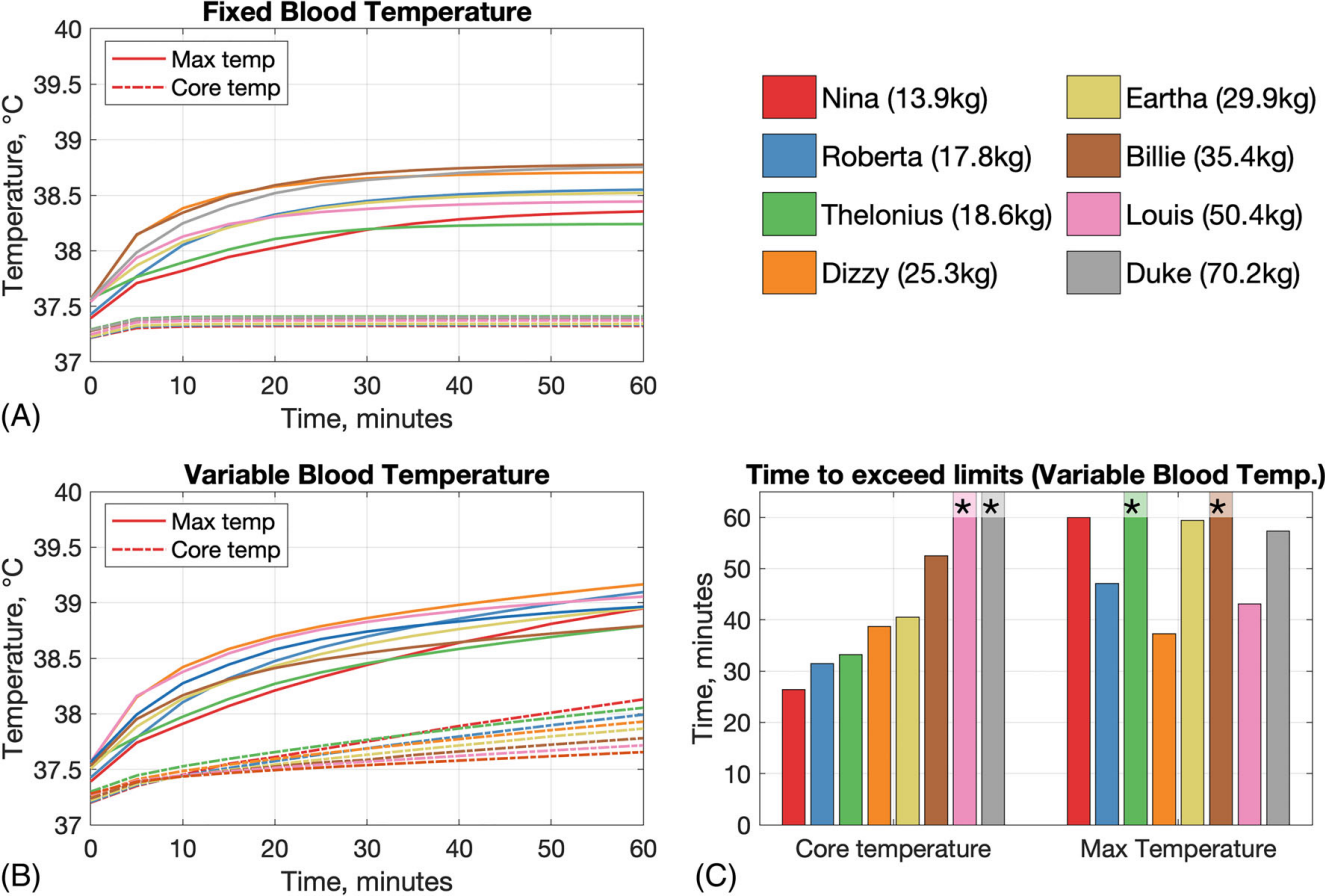
Summary of results from thermal simulations, run at power level and resulting in hdSAR = 3.2 W kg^−1^ for each model. Top row: Results for fixed blood temperature; bottom row: results for variable blood temperature. Solid lines in (A) and (B) indicate overall maximum temperature, and dashed lines indicate core temperature; the latter is defined as the average over the heart and brain. (A) With fixed blood temperature, after 1 hour of exposure, the maximum temperature does not exceed 39°C and core temperature increases are about 0.1°C, for all models. (B) When blood temperature is variable, IEC guidelines for core (change of 0.5°C) and/or maximum local temperature (39°C) are exceeded for most models. (C) Times to exceed IEC guidelines for the data presented in (B). Asterisks indicate that the limit was not exceeded. In general, for the smaller models, core temperature limit is exceeded before maximum temperature limit

Figure 5 illustrates the results for simulations for fixed (Figure 5A) and variable (Figure 5B,C) blood temperature. For the simulations assuming a fixed blood temperature, none of the models exceeded the maximum temperature within 60 min, and core temperature increases were limited to about 0.1°C for all models. However, if blood temperature is allowed to vary, then core temperatures increase and exceed a change of 0.5°C (the IEC limit) in under 60 min for all models with mass ≤ 35.4 kg; the time to exceed the limit increases monotonically with mass. Maximum local temperatures also exceed 39°C in most of these models within 60 min, although there is no clear relationship between mass and time taken to exceed limits in this case. Supporting Information Figure S3 explores relationships between the change in peak ($\Delta T_{\max}$) and core ($\Delta T_{\text{core}}$) temperatures over 60 min and the model mass, psSAR_10g_ and wbSAR (all thermal simulations were run at a fixed hdSAR = 3.2 W kg^−1^). There is a clear linear relationship between wbSAR and $\Delta T_{\text{core}}$ for the variable blood temperature case, with the following form:

(6) $$\Delta T_{\text{core}}=0.11+1.02\;\text{wbSAR}\;\left(R^{2}=0.99\right)$$

**FIGURE 6:**
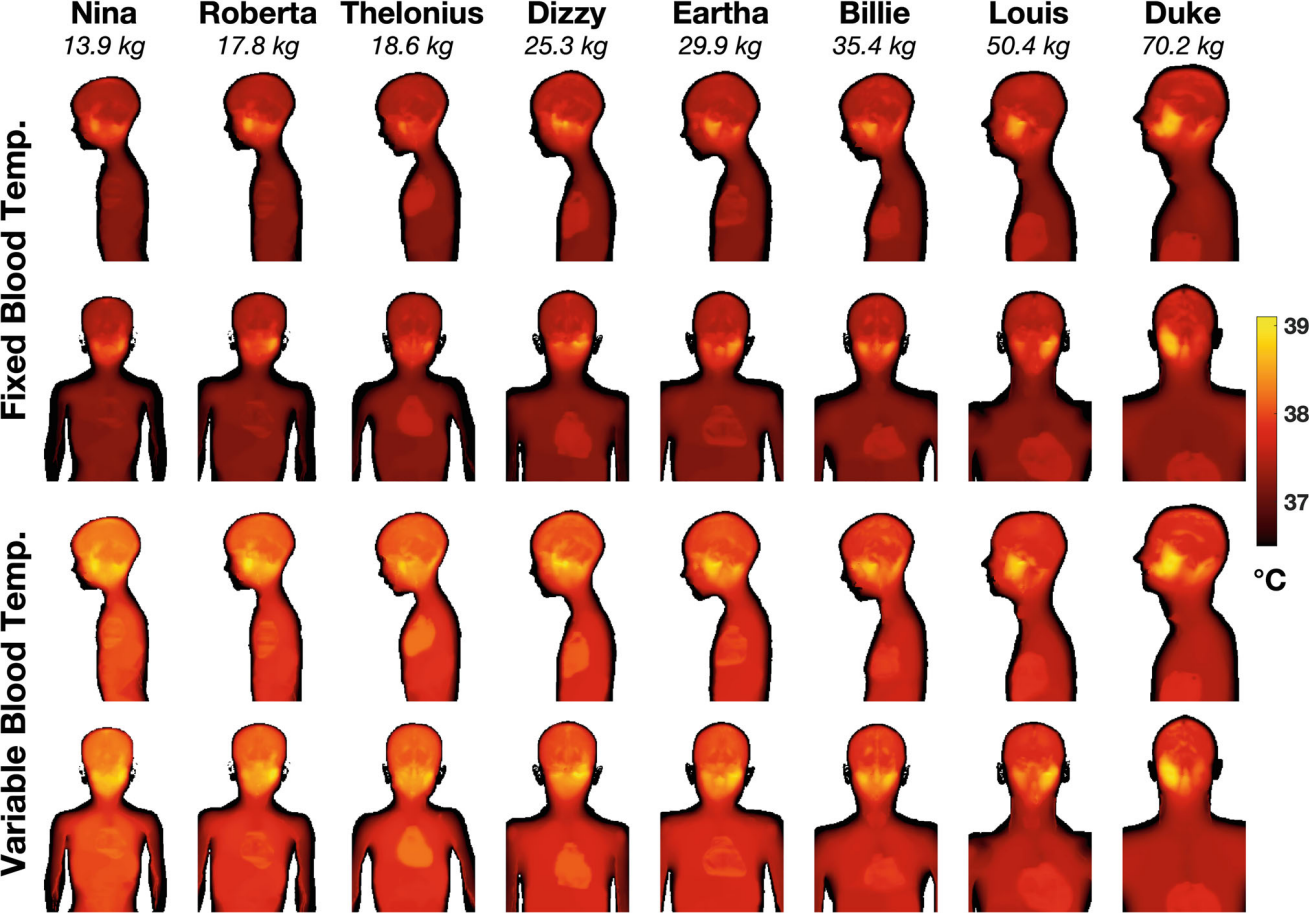
Maximum projections of temperature after 60 min exposure at hdSAR = 3.2 W kg^−1^ for thermal calculations with fixed blood temperature (top two rows) and variable blood temperature (lower two rows). Within each block, the upper row is a sagittal projection, and the lower row is coronal. Fixed blood temperature leads to generally lower temperatures than when variable blood temperature is modeled, but the spatial distributions are similar

If blood temperatures are fixed, then $\Delta T_{\text{core}}$ remains small and not correlated with any parameter. The value of $\Delta T_{\max}$ appeared uncorrelated with investigated parameters except the case of fixed blood temperature when a correlation of the form

(7) $$\Delta T_{\max}=-0.37+0.162\;{\text{psSAR}}_{10g}\;\left(R^{2}=0.66\right)$$

was observed. Figure 6 shows maximum projections of the temperature distributions in all models after 60 min of RF exposure at maximum hdSAR = 3.2 W kg^−1^. In general, we observe that variable blood temperature simulations give higher temperatures than fixed blood temperatures, but that the spatial temperature distributions are similar in both cases. Higher temperatures were predicted in inferior–anterior regions of the head, in contrast to the SAR distributions shown in Figure 3. These higher temperatures occurred in tissues with lower perfusion, such as skull, cartilage, mandible, and CSF.

## 4 DISCUSSION

This safety assessment for MRI at 7 T of children with ages and masses in the ranges of 3–14 years and 13.9–50.8 kg, respectively, considered EM and thermal simulations of RF exposure at 297 MHz within a single‐channel birdcage head transmit coil designed for adults. Seven child models from the Virtual Population^15^ were used, and results of simulations were compared with those obtained using the adult male model Duke. Recent CE and FDA 510(k) certifications^1^ for a 7T head transmit coil allow clinical use with mass > 30 kg; this study included five models < 30 kg for comparison, although the coil model used in this work did not come from the manufacturer of that device.

### 4.1 Effect of body size on EM simulations

EM simulations suggested that SAR would generally be elevated in the smaller child models when compared with adult or larger child models. In particular, negative linear correlations with mass were observed for hdSAR; correlations between mass and psSAR_10g_ were not observed. Using the fitted linear relationship, we expect hdSAR to increase by 30% when body mass reduces from 70 kg to 30 kg. Further reducing the mass from 30 kg to 15 kg would result in an additional approximate 9% increase in hdSAR.

Correlation between hdSAR and mass is relevant, as hdSAR is predicted to be the limiting safety constraint for all models for the head coil simulated in this work (Figure 2B). It remained the limiting factor when spatially shifting the smaller models in the AP and RL directions; however, SI shifts caused psSAR_10g_ to become the limiting factor in some cases. Other published reports have also found the ratio between psSAR_10g_ and hdSAR to be in a similar range for 7T head transmit coils. For example, Restivo et al^34^ found this ratio to be in the range of 2.9–3.7 for four “patient based” models derived by blending the Duke model with patient data. Van Lier et al^35^ reported a ratio of 3.8 for Duke. In the current study, the ratio for Duke located with corpus callosum centered in the coil was 3.0, although an inferior axial shift of the model of 25 mm increased this to 3.9. Our own recent study of neonatal exposure to the same head coil model^4^ also found hdSAR to be the limiting factor. In another study, Fontana et al^5^ described EM simulations of an adult female, Dizzy, and Billie head centered within a longer (275 mm) and smaller diameter (295 mm) 7T volume head coil and found that maximum local SAR within the head was higher in the children (47% for Billie and 39% for Dizzy) compared with that in the adult female.

The sensitivity of SAR estimation to pose has been studied in depth by Kopanoglu et al^36^ in the context of parallel transmit (with an adult model). Considering only the CP mode of their head coil, which could be considered similar to the birdcage used in this study, they report hdSAR changing by < 5% over a range of shifts ≤20mm, whereas psSAR_10g_ could change up to 2.1 fold. They also report much greater sensitivity to shifts in AP and LR directions than SI. In fact, when considering only the Duke model and when considering SAR normalized to net power, and not $B_{1}^{+}$, our results are similar with hdSAR only changing by more than 5% for a −50‐mm SI shift, and in this case the change was a reduction. We also observed much smaller changes in psSAR_10g_, with only −25‐mm and −50‐mm shifts in the SI direction resulting in changes over 10%. This difference may reflect the different coil designs used in these studies. We also note that adjusting the power to account for differences in $B_{1}^{+}$ caused much greater position dependence of SAR in our study.

### 4.2 Age dependence of EM properties and uncertainty

Because the tissues of young children are expected to have higher water content than those of an adult,^37^ the potential impact on dielectric properties of young tissues and the consequent effect on $B_{1}^{+}$ and SAR were investigated. Based on the Dizzy model, which was predicted to have the highest total body water per kilogram of the models considered here, increases in the range of about 8%–15% for both permittivity and conductivity for bone marrow, white matter, cartilage, eye lens, and skin, relative to adult values, were predicted. For tissues with low water content (bone, fat, mandible, and skull), the increases were about 25%. Smaller changes of 3%–4% were predicted for the remaining tissues. The effect of these increases on the power budget parameters, mean $B_{1}^{+}$ (in the central axial slice), and SAR were small (< 5%).In light of other uncertainties such as the measurement error in tissue dielectric properties (6%–10% according to Gabriel et al^38^), estimating water content of children's tissues (∼16% from Wells et al^16^), and errors associated with finite‐difference time‐domain simulations (eg, < 5% grid‐dependent variability; see Supporting Information Figure S4), there is no strong case for using age‐adjusted parameters in the group of child models investigated. This is in contrast with neonates, in whom more significant differences in dielectric properties are expected.^4^

### 4.3 Thermal simulations

The thermal simulations reported here were based on a number of conservative assumptions, including use of adult values for thermal properties of tissues, a stable core temperature in the absence of RF (when children may actually cool down in the MR environment^30^), and heat loss being determined by a single heat transfer coefficient at the skin/environment boundary without considering other mechanisms such as evaporation. Furthermore, continuous RF exposure at the maximum permitted power level for each model was assumed; in practice, the time‐averaged duty cycle over an entire examination will be < 100%. With these assumptions in place, but assuming fixed blood temperature (which is common for MRI safety assessments), continuous RF exposure at hdSAR = 3.2 W kg^−1^ resulted in peak and core temperature changes that did not exceed IEC guidelines. Holding a fixed blood temperature is equivalent to assuming that unspecified thermoregulation processes counteract the systemic effect of any heat stimulus. If this assumption is removed by allowing variable blood temperature, the limit of 0.5°C core temperature increase^9^ was exceeded in under 60 min for all models ≤ 35.4 kg, and this happened before the maximum recommended temperature of 39°C^9^ is exceeded for all of these except Dizzy, when the time taken is comparable.

Use of variable blood temperature was the major factor in the elevated temperatures seen in these simulations, and is in line with results from Hirata et al,^28^ who also found that local temperatures are strongly impacted by allowing the blood temperature within the bioheat equation to vary. Hirata et al found that simulated blood temperature changes in children and adults broadly agreed with experimental measures, although the time profiles of changes were not always well predicted. They found that the key driver of blood temperature change was the wbSAR, in agreement with our finding of a linear relationship. Equation (6) predicts that core temperature changes by approximately 1°C per W kg^−1^ of wbSAR, which are greater than the values observed by Hirata et al. This may be because our model excluded important heat loss mechanisms, such as sweating, which were shown to have a significant effect in practice,^39^ or indeed because of the use of different RF frequencies between studies. Furthermore, because Equations (6) and (7) relate temperature changes to wbSAR and psSAR_10g_ for different models each exposed at maximum hdSAR, they should not be interpreted as temperature versus exposure for a single model. There is currently mixed evidence on how core temperatures do change during MRI examinations: a recent review^40^ cited several reports in which increases over 0.5°C were observed. There are also reports on temperature of sedated children undergoing MRI: results are mixed, with some showing temperature decreases in smaller children,^41^ some with larger increases up to 0.6°C–0.7°C,^42^ and some with no significant change.^31^ At present we do not have sufficient evidence to confirm the thermal predictions made in this work; we aim to build this up in future studies by recording measured temperatures of children undergoing MRI.

### 4.4 Study limitations

This study looked at a range of human body models but considered only one RF coil model, as the objective was to examine variability due to human body size specifically for a coil of this design. Therefore, the results cannot necessarily be directly translated to other coils in which design‐specific factors influencing SAR distributions may be important. This said, we did observe that in general SAR hotspots appeared to be primarily correlated with anatomy (usually in the cortex in the superior part of the brain; see Figure 3). Furthermore, the main limiting SAR measure was found to be hdSAR in all cases, which is less dependent on small features of the coil. Nevertheless, it cannot be ruled out that other designs would have more specific local effects (eg, hotspots near coil tuning elements); in particular, these may alter the ratio between psSAR_10g_ and hdSAR, and this should be the study of future work. Similarly, future research would need to investigate the use of parallel transmit technology, as this study focused only on a birdcage coil driven in CP mode.

## 5 CONCLUSIONS

This study of a 297‐MHz birdcage design head‐transmit coil with a range of models (age 3–34, mass 14–70 kg) identified a linear relationship between hdSAR and body mass. When centered in the coil, the hdSAR was found to be the limiting factor for all models, with the relative importance of the hdSAR compared with psSAR_10g_ increasing as body mass decreases. These results are sensitive to positioning within the birdcage coil, particularly when $B_{1}^{+}$ is accounted for. The smaller child models (≤ 35.4 kg) are less sensitive than the adult to LR and AP shifts. All models are most sensitive to SI shifts. Careful positioning and restraint of head movement could be used to reduce uncertainty.

In light of positional uncertainties, both hdSAR and psSAR_10g_ and their relation with patient size should be considered for safety assessments. An important practical factor is that within the IEC guidelines, although the psSAR_10g_ limit can be doubled when moving to “first level” control mode, the hdSAR limit remains at 3.2 W kg^−1^.^9^

Thermal modeling based on arguably conservative assumptions (including allowing variable blood temperature, indicative of poor thermoregulation) suggests that core temperature increases will exceed 0.5°C under continuous exposure to hdSAR of 3.2 W kg^−1^ within 1 hour, with the smallest subject (“Nina,” 13.9 kg) exceeding this after 26 min. However, use of fixed blood temperature (as is common for MRI studies) finds that temperature limits, both in terms of core and local increases, are not exceeded within 60 min. There is insufficient evidence to decide conclusively which of these predictions is more likely; however, an important conclusion of this work is that the main thermal risk is likely to be systemic rather than local heating. This can be effectively mitigated by active monitoring of core temperature during MRI examinations, and such measurements may also prove valuable for validating and further calibrating future predictions of this type.

## Supporting information


**Figure S1** Extreme axial positions (z = −50 mm left and z = 50 mm right) of Eartha within head coil
**Figure S2** Ratio of peak‐spatial 10*g* averaged specific absorption rate (psSAR_10g_) to head average SAR (hdSAR) as a function of subject position. The shading on the plot background illustrates whether the limiting quantity is hdSAR (pink) or psSAR_10g_ (yellow). For the unshifted models, the hdSAR is always the limiting value (see Figure 2B). For anterior–posterior (AP) and left–right (LR) shifts, this is also true for the smaller models, but not for Louis or Duke. If the models are shifted in the superior–inferior (SI) direction, then in general the psSAR_10g_ becomes the limiting value
**Figure S3** Top row: The psSAR_10g_ and whole body averaged SAR (wbSAR) applied during thermal simulations of each model; these simulations were run at a fixed hdSAR = 3.2 W kg^−1^, which meant that the other SAR parameters varied. The wbSAR was much greater in the smaller models, as would be expected because the head is a larger fraction of body mass, but was much less than the 2 W kg^−1^ International Electrotechnical Commission (IEC) limit in all cases. Remaining rows: The temperature increase after 60 min of simulated RF exposure (change in maximum temperature $\Delta T_{\max}$ in left column and change in core temperature $\Delta T_{\text{core}}$ in right column) as a function of the subject mass and different SAR metrics. In each case, the blue circles represent simulations with fixed blood temperature and red triangles represent variable blood temperature. We looked for linear correlations among all variables; the quoted R^2^ values on each plot represent the quality of a linear fit to the data (some relations are clearly nonlinear, in which case the quoted R^2^ does not reflect the true strength of correlation). For the cases in which $R^{2}\geq 0.5$, linear trend lines are also plotted (dashed lines). Peak temperature $\Delta T_{\max}$ was not observed to strongly correlate with any of the parameters, especially when variable blood temperature was used. In the case of fixed blood temperature, a weak (R^2^ = 0.66) correlation was observed between psSAR_10g_ and $\Delta T_{\max}$. This may indicate that peak local temperatures are driven by elevated local SAR, although this is contradicted by Figures 3 and 6, which indicate that the maxima in SAR and temperature change are generally not colocated. Furthermore, this trend becomes significantly weaker (R^2^ = 0.44) if the “Thelonius” data set, for which psSAR_10g_ was particularly low, is excluded. Stronger trends were seen for $\Delta T_{\text{core}}$ when using variable blood temperature (but not at all for fixed blood temperature, as this tends to hold core temperature constant). The value of $\Delta T_{\text{core}}$ has an almost perfectly linear relationship with wbSAR (R^2^ = 0.99); there is also a strong correlation with mass, but this is probably because mass and wbSAR are themselves closely related. A negative trend between psSAR_10g_ and $\Delta T_{\text{core}}$ was also observed, which does not make physical sense on its own, but is most likely caused by the correlation between $\Delta T_{\text{core}}$ and wbSAR, as wbSAR and psSAR_10g_ were not varied independently in this study. Note that Equations (6) and (7) should be considered valid only over the range of SAR values simulated. Indeed, they correspond to the variation in temperature as a function of wbSAR and psSAR_10g_ for different models, each exposed at maximum hdSAR, not different exposures of the same model. Hence, extrapolation to “zero exposure” of either metric is unrealistic in this context
**Figure S4** Grid refinement study for the Dizzy model. Black circles indicate grid size used for the rest of the paper. Results are stable across grid size, and the selected grid gives comparable results to more detailed grids (more cells)
**Table S1** Details of grid settings for electromagnetic simulations
**Table S2** Volumes used to calculate hdSAR
**Table S3** Age, mass, and height used to calculate total body water W_T_ according to Equation (1)
**Table S4** Blood volume assuming 75 ml kg^−1^ for children^13^ and 70 ml kg^−1^ for the adult^14^
*Note*: These blood volumes were used for simulating blood temperature increase, but do not relate to the perfusion term included in the bioheat model. This is because the model teats the latter as a tissue‐specific loss mechanism but does not conserve energy transport around the body.
**Table S5.1** Power budget, mean $B_{1}^{+}$, and SARs for Nina model (13.9 kg) shifted within the birdcage coil. Relative position is shift (in millimeters) along the specified axis relative to the original brain centered position (x = 0, y = 0, z = 0).
The mean is calculated within the same anatomical slice (ie, the slice shifts as the Nina model shifts), thereby simulating imaging of the same anatomy with an altered positioning of the subject. A ratio psSAR_10g_/hdSAR < 3.125 indicates that the hdSAR limit (3.2 W kg^−1^) is more conservative than the psSAR_10g_ limit (10 W kg^−1^); limits as specified by the IEC.^9^
**Table S5.2** Power budget, mean $B_{1}^{+}$, and SARs for Roberta model (17.8 kg) shifted within the birdcage coil
**Table S5.3** Power budget, mean $B_{1}^{+}$, and SARs for Thelonius model (18.6 kg) shifted within the birdcage coil
**Table S5.4** Power budget, mean $B_{1}^{+}$, and SARs for Dizzy model (25.3 kg) shifted within the birdcage coil
**Table S5.5** Power budget, mean $B_{1}^{+}$, and SARs for Eartha model (29.9 kg) shifted within the birdcage coil
**Table S5.6** Power budget, mean $B_{1}^{+}$, and SARs for Billie model (35.4 kg) shifted within the birdcage coil
**Table S5.7** Power budget, mean $B_{1}^{+}$, and SARs for Louis model (50.4 kg) shifted within the birdcage coil
**Table S5.8** Power budget, mean $B_{1}^{+}$, and SARs for Duke model (70.2 kg) shifted within the birdcage coil

## Data Availability

Full summary data from EM and thermal simulations are provided in the tables and supporting tables of this article. The virtual family models are available under license from Zurich Med Tech and so cannot be shared openly, however more detailed results can be obtained from the corresponding author on reasonable request
