# Supplementary material for: Evaluation of specific absorption rate and heating in children exposed to a 7T MRI head coil
Source: Magn Reson Med. 2022 Jun 6;88(3):1434–49. doi: 10.1002/mrm.29283 (PMC9328145; doi:10.1002/mrm.29283)
Supplement: Supplementary file 1 — Figure S1 Extreme axial positions (z = −50 mm left and z = 50 mm right) of Eartha within head coil Figure S2 Ratio of peak‐spatial 10g averaged specific absorption rate (psSAR10g) to head average SAR (hdSAR) as a function of subject position. The shading on the plot background illustrates whether the limiting quantity is hdSAR (pink) or psSAR10g (yellow). For the unshifted models, the hdSAR is always the limiting value (see Figure 2B). For anterior–posterior (AP) and left–right (LR) shifts, this is also true for the smaller models, but not for Louis or Duke. If the models are shifted in the superior–inferior (SI) direction, then in general the psSAR10g becomes the limiting value Figure S3 Top row: The psSAR10g and whole body averaged SAR (wbSAR) applied during thermal simulations of each model; these simulations were run at a fixed hdSAR = 3.2 W kg−1, which meant that the other SAR parameters varied. The wbSAR was much greater in the smaller models, as would be expected because the head is a larger fraction of body mass, but was much less than the 2 W kg−1 International Electrotechnical Commission (IEC) limit in all cases. Remaining rows: The temperature increase after 60 min of simulated RF exposure (change in maximum temperature ΔTmax in left column and change in core temperature ΔTcore in right column) as a function of the subject mass and different SAR metrics. In each case, the blue circles represent simulations with fixed blood temperature and red triangles represent variable blood temperature. We looked for linear correlations among all variables; the quoted R2 values on each plot represent the quality of a linear fit to the data (some relations are clearly nonlinear, in which case the quoted R2 does not reflect the true strength of correlation). For the cases in which R2≥0.5, linear trend lines are also plotted (dashed lines). Peak temperature ΔTmax was not observed to strongly correlate with any of the parameters, especially when variable blood temperature [file MRM-88-1434-s001.docx]

Supporting Information

This document contains four supporting figures and twelve tables; the tables summarize the results of all simulations and can be used to recreate the electromagnetic simulation results presented in the paper.


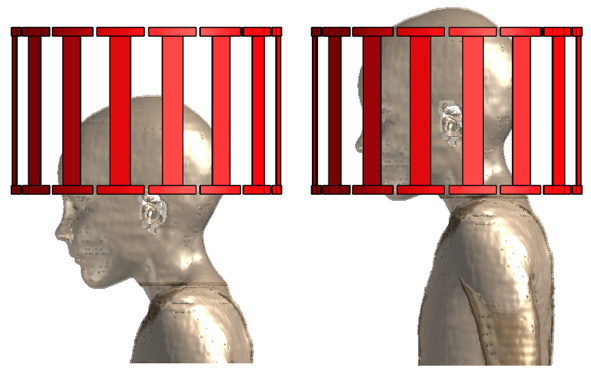


**Figure S1** Extreme axial positions (z=-50 mm left and z=50 mm right) of Eartha within head coil.


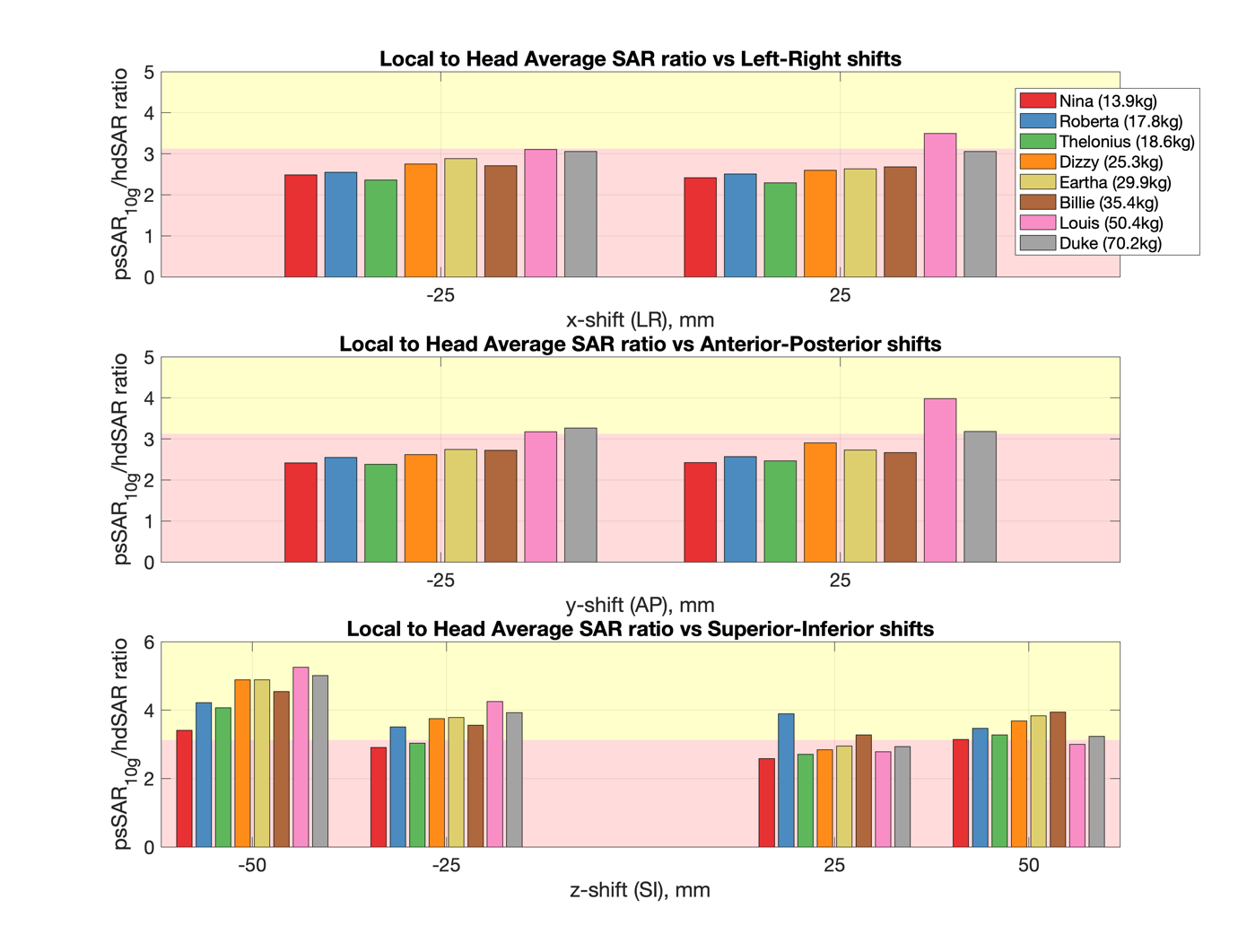


**Figure S2:** Ratio of psSAR_10g_ to hdSAR as a function of subject position. The shading on the plot background illustrates whether the limiting quantity is head averaged SAR (pink) or psSAR_10g_ (yellow). For the unshifted models the head average SAR is always the limiting value (see figure 2B). For AP and LR shifts this is also true for the smaller models, but not for Louis or Duke. If the models are shifted in the SI direction then in general the psSAR_10g_ becomes the limiting value.


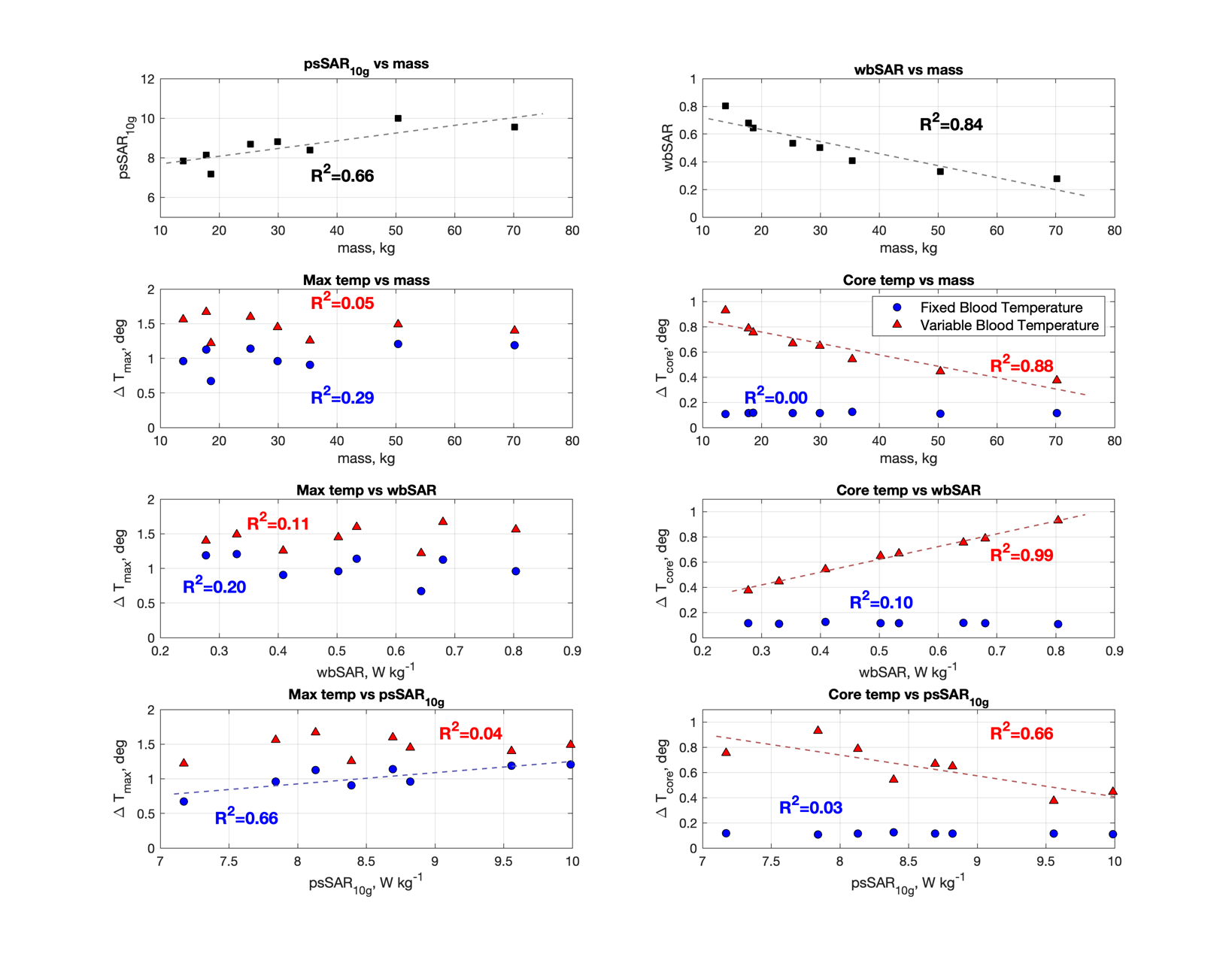


**Figure S3: Top Row** shows psSAR_10g_ and wbSAR applied during thermal simulations of each model – these simulations were run at a fixed hdSAR = 3.2 W kg^-1^ which meant that the other SAR parameters varied. Whole body averaged SAR was much greater in the smaller models, as would be expected since the head is a larger fraction of body mass, but was much less than the 2 W kg^-1^ IEC limit in all cases. **Remaining rows** examine the temperature increase after 60 minutes of simulated RF exposure (change in maximum temperature $\Delta T_{max}$ in left column and change in core temperature $\Delta T_{core}$ in right column) as a function of the subject mass and different SAR metrics. In each case the blue circles represent simulations with fixed blood temperature and red triangles are variable blood temperature. We looked for linear correlations between all variables; the quoted R^2^ values on each plot represent the quality of a **linear** fit to the data (some relations are clearly non-linear, in which case the quoted R^2^ doesn’t reflect the true strength of correlation). For the cases where $R^{2}\geq0.5$ linear trend lines are also plotted (dashed lines). Peak temperature $\Delta T_{max}$ was not observed to strongly correlate with any of the parameters, especially when variable blood temperature was used. In the case of fixed blood temperature a weak (R^2^=0.66) correlation was observed between psSAR_10g_ and $\Delta T_{max}$. This may indicate that peak local temperatures are driven by elevated local SAR, though this is contradicted by Figures 3 and 6 which indicate that the maxima in SAR and temperature change are generally not co-located. Furthermore this trend becomes significantly weaker (R^2^=0.44) if the ‘Thelonius’ dataset, for which psSAR_10g_ was particularly low, is excluded. Stronger trends were seen for $\Delta T_{core}$ when using variable blood temperature (but not at all for fixed blood temperature as this tends to hold core temperature constant). $\Delta T_{core}$ has an almost perfectly linear relationship with wbSAR (R^2^=0.99); there is also a strong correlation with mass, but this is probably because mass and wbSAR are themselves closely related. A negative trend between psSAR_10g_ and $\Delta T_{core}$ was also observed, which doesn’t make physical sense on its own, but is most likely caused by the correlation between $\Delta T_{core}$ and wbSAR since wbSAR and psSAR_10g_ were not varied independently in this study. Note that Equations 6 & 7 should be considered valid only over the range of SAR values simulated. Indeed, they correspond to the variation in temperature as a function of wbSAR and psSAR_10g_ for different models each exposed at maximum hdSAR, not different exposures of the same model. Hence, extrapolation to ‘zero exposure’ of either metric is unrealistic in this context.

# Gridding and convergence

The Sim4life “non-homogeneous intelligent grid” functionality was used, with minimum and maximum steps and total grid size (number of cells) shown in Table S1. Simulation times were set to 100 periods, in practice this led to convergence of field patterns within the range -43 to -50dB dependent on the model. Since the Sim4life gridding produces variable density grids, step sizes are spatially variable. We investigated sensitivity to grid properties by changing maximum step size and re-gridding a few times for the Dizzy model. Figure S4 plots metrics of RF power, B_1_^+^ and SAR as a function of the total grid size (we use total grid size here as the mesh density is non-uniform so total number of cells gives a good reflection of the grid complexity). The figure shows that the results remain stable when grids are refined further (i.e. larger number of cells) than the values used in this study; absorbed and radiated power, wbSAR, hdSAR and B_1_^+^ vary ≤3% while peak spatial SAR varies by ≤5%. This indicates that the results are not highly sensitive to the grid.

| **Simulation** | **Minimum step (mm)** | | | **Maximum step (mm)** | | | **Number of cells**  **(x 10^6^)** |
| --- | --- | --- | --- | --- | --- | --- | --- |
|  | **x** | **y** | **z** | **x** | **y** | **z** |  |
| Nina | 0.69 | 0.69 | 1.0 | 50.3 | 66.2 | 64.7 | 25.4 |
| Roberta | 0.69 | 0.69 | 1.0 | 59.5 | 48.0 | 59.9 | 31.7 |
| Thelonius | 0.69 | 0.69 | 1.0 | 60.0 | 66.2 | 64.7 | 35.3 |
| Dizzy | 0.69 | 0.69 | 1.0 | 59.1 | 48.0 | 59.9 | 45.2 |
| Eartha | 0.69 | 0.69 | 1.0 | 63.0 | 66.2 | 64.7 | 46.9 |
| Billie | 0.69 | 0.69 | 1.0 | 63.1 | 66.2 | 64.7 | 51.5 |
| Louis | 0.69 | 0.63 | 1.0 | 57.3 | 66.2 | 64.7 | 73.9 |
| Duke | 0.69 | 0.69 | 1.0 | 48.5 | 66.2 | 64.7 | 70.0 |

**Table S1.** Details of grid settings for EM simulations


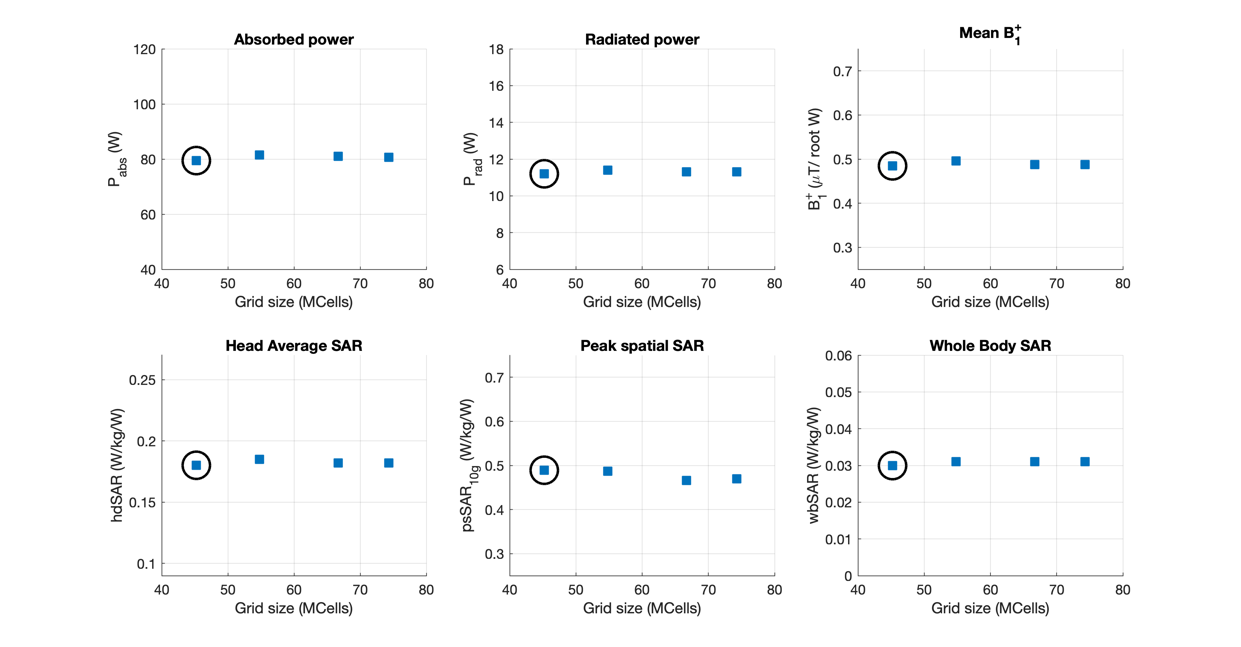


**Figure S4:** Grid refinement study for Dizzy model. Black circles indicate grid size used for the rest of the paper. Results are stable across grid size, and the selected grid gives comparable results to more detailed grids (more cells).

# Supporting data for thermal models

|  | **Nina** | **Roberta** | **Thelonius** | **Dizzy** | **Eartha** | **Billie** | **Louis** | **Duke** |
| --- | --- | --- | --- | --- | --- | --- | --- | --- |
| Head volume (cm^3^) | 2392 | 2769 | 2964 | 3350 | 3545 | 3310 | 3968 | 4800 |

**Table S2.** Volumes used to calculate head average SAR.

|  | **Nina** | **Roberta** | **Thelonius** | **Dizzy** | **Eartha** | **Billie** | **Louis** | **Duke** |
| --- | --- | --- | --- | --- | --- | --- | --- | --- |
| Age (years) | 3 | 5 | 6 | 8 | 8 | 11 | 14 | 34 |
| Mass (kg) | 13.9 | 17.8 | 18.6 | 25.3 | 29.9 | 35.4 | 50.8 | 70.2 |
| Height (m) | 0.92 | 1.09 | 1.16 | 1.37 | 1.36 | 1.46 | 1.68 | 1.77 |
| W_T_/mass (l/kg) | 0.60 | 0.62 | 0.65 | 0.66 | 0.57 | 0.58 | 0.58 | 0.59 |

**Table S3**. Age, mass and height used to calculate total body water W_T_ according to Equation [1].

|  | **Nina** | **Roberta** | **Thelonius** | **Dizzy** | **Eartha** | **Billie** | **Louis** | **Duke** |
| --- | --- | --- | --- | --- | --- | --- | --- | --- |
| Blood volume (l) | 1.043 | 1.335 | 1.395 | 1.898 | 2.243 | 2.655 | 3.780 | 4.914 |

**Table S4**. Blood volume assuming 75 ml/kg for children (13) and 70 ml/kg for the adult (14). Note that these blood volumes were used for simulating blood temperature increase, but do not relate to the perfusion term included in the bioheat model. This is because the model treats the latter as a tissue specific loss mechanism but doesn’t conserve energy transport around the body.

# Supplementary results: full data for all models

|  | | **NINA** | | | | | | | |
| --- | --- | --- | --- | --- | --- | --- | --- | --- | --- |
| **Relative position** | | **x-25** | **x+25** | **y-25** | **y+25** | **z-50** | **z-25** | **z+25** | **z+50** |
| **Power budget (%)** | Absorbed | 74.1 | 71.7 | 75.0 | 71.7 | 59.3 | 67.9 | 74.3 | 72.9 |
|  | Reflected/Coupled | 4.4 | 5.2 | 3.1 | 5.5 | 14.7 | 7.9 | 3.9 | 4.4 |
|  | Radiated | 17.2 | 18.5 | 17.4 | 18.3 | 21.3 | 19.8 | 16.8 | 15.9 |
|  | Other | 4.3 | 4.6 | 4.5 | 4.5 | 4.7 | 4.4 | 5.0 | 6.8 |
| **Mean B_1_^+^ for 1 W total power (**$\boldsymbol{\mu}$**T/√W)** | | 0.542 | 0.528 | 0.539 | 0.533 | 0.550 | 0.566 | 0.470 | 0.383 |
| **SAR per W total power (W/kg/W)** | Head average | 0.215 | 0.207 | 0.215 | 0.209 | 0.159 | 0.194 | 0.208 | 0.187 |
|  | psSAR_10g_ | 0.535 | 0.500 | 0.520 | 0.507 | 0.543 | 0.565 | 0.538 | 0.587 |
|  | Body average | 0.052 | 0.052 | 0.054 | 0.052 | 0.043 | 0.049 | 0.054 | 0.053 |
| **SAR per mean B_1_^+^ (W/kg/**$\boldsymbol{\mu}$**T^2^)** | Head average | 0.732 | 0.743 | 0.740 | 0.736 | 0.526 | 0.606 | 0.942 | 1.275 |
|  | psSAR_10g_ | 1.821 | 1.794 | 1.790 | 1.785 | 1.795 | 1.764 | 2.435 | 4.002 |
|  | Body average | 0.177 | 0.187 | 0.186 | 0.183 | 0.142 | 0.153 | 0.244 | 0.361 |
| **Ratio psSAR_10g_/hdSAR** | | 2.49 | 2.42 | 2.42 | 2.43 | 3.42 | 3.38 | 2.59 | 3.14 |

**Table S5.1** Power budget, mean B_1_^+^ and SARs for Nina model shifted within the birdcage coil. Relative position is shift (in mm) along the specified axis relative to the original brain centered position (x=0, y=0, z =0). The mean is calculated within the same anatomical slice, i.e. the slice shifts as the Nina model shifts, thereby simulating imaging of the same anatomy with an altered positioning of the subject. A ratio psSAR_10g_/head average SAR < 3.125 indicates that the head average SAR limit (3.2 $Wkg^{-1}$) is more conservative than the psSAR_10g_ limit (10 $Wkg^{-1}$); limits as specified by the IEC (9).

|  | | **ROBERTA** | | | | | | | |
| --- | --- | --- | --- | --- | --- | --- | --- | --- | --- |
| **Relative position** | | **x-25** | **x+25** | **y-25** | **y+25** | **z-50** | **z-25** | **z+25** | **z+50** |
| **Power budget (%)** | Absorbed | 78.2 | 78.8 | 79.5 | 77.2 | 64.8 | 73.0 | 79.2 | 77.4 |
|  | Reflected/Coupled | 3.5 | 1.4 | 1.7 | 2.2 | 13.5 | 6.0 | 2.3 | 4.6 |
|  | Radiated | 14.0 | 15.4 | 14.3 | 15.2 | 17.4 | 16.3 | 14.1 | 13.6 |
|  | Other | 4.3 | 4.4 | 4.5 | 5.4 | 4.3 | 4.7 | 4.4 | 4.4 |
| **Mean B_1_^+^ for 1 W total power (**$\boldsymbol{\mu}$**T/√W)** | | 0.506 | 0.509 | 0.509 | 0.504 | 0.513 | 0.536 | 0.434 | 0.334 |
| **SAR per W total power (W/kg/W)** | Head average | 0.209 | 0.214 | 0.214 | 0.210 | 0.167 | 0.196 | 0.201 | 0.175 |
|  | psSAR_10g_ | 0.533 | 0.536 | 0.545 | 0.540 | 0.705 | 0.688 | 0.783 | 0.607 |
|  | Body average | 0.044 | 0.045 | 0.045 | 0.044 | 0.037 | 0.041 | 0.045 | 0.044 |
| **SAR per mean B_1_^+^ (W/kg/**$\boldsymbol{\mu}$**T^2^)** | Head average | 0.816 | 0.826 | 0.826 | 0.827 | 0.635 | 0.682 | 1.067 | 1.569 |
|  | psSAR_10g_ | 2.082 | 2.069 | 2.104 | 2.126 | 2.679 | 2.395 | 4.157 | 5.441 |
|  | Body average | 0.172 | 0.174 | 0.174 | 0.173 | 0.141 | 0.143 | 0.239 | 0.394 |
| **Ratio psSAR_10g_/ hdSAR** | | 2.55 | 2.50 | 2.55 | 2.57 | 4.22 | 3.51 | 3.90 | 3.47 |

**Table S5.2** Power budget, mean B_1_^+^ and SARs for Roberta model shifted within the birdcage coil.

|  | | **THELONIUS** | | | | | | | |
| --- | --- | --- | --- | --- | --- | --- | --- | --- | --- |
| **Relative position** | | **x-25** | **x+25** | **y-25** | **y+25** | **z-50** | **z-25** | **z+25** | **z+50** |
| **Power budget (%)** | Absorbed | 80.5 | 78.2 | 80.4 | 77.8 | 64.1 | 73.3 | 80.1 | 79.6 |
|  | Reflected/Coupled | 2.2 | 2.7 | 2.6 | 3.4 | 15.5 | 7.2 | 2.8 | 3.1 |
|  | Radiated | 13.2 | 15.0 | 13.2 | 14.8 | 16.3 | 15.6 | 13.3 | 12.4 |
|  | Other | 4.1 | 4.1 | 3.8 | 4.0 | 4.1 | 3.9 | 3.8 | 4.9 |
| **Mean B_1_^+^ for 1 W total power (**$\boldsymbol{\mu}$**T/√W)** | | 0.500 | 0.495 | 0.499 | 0.491 | 0.506 | 0.529 | 0.419 | 0.326 |
| **SAR per W total power (W/kg/W)** | Head average | 0.201 | 0.197 | 0.203 | 0.194 | 0.156 | 0.186 | 0.195 | 0.175 |
|  | psSAR_10g_ | 0.474 | 0.452 | 0.483 | 0.479 | 0.635 | 0.565 | 0.529 | 0.574 |
|  | Body average | 0.041 | 0.040 | 0.041 | 0.040 | 0.033 | 0.038 | 0.042 | 0.041 |
| **SAR per mean B_1_^+^ (W/kg/**$\boldsymbol{\mu}$**T^2^)** | Head average | 0.804 | 0.804 | 0.815 | 0.805 | 0.609 | 0.665 | 1.111 | 1.647 |
|  | psSAR_10g_ | 1.896 | 1.845 | 1.940 | 1.987 | 2.480 | 2.019 | 3.013 | 5.401 |
|  | Body average | 0.164 | 0.163 | 0.165 | 0.166 | 0.129 | 0.136 | 0.239 | 0.386 |
| **Ratio psSAR_10g_/ hdSAR** | | 2.36 | 2.30 | 2.38 | 2.47 | 4.07 | 3.04 | 2.71 | 3.28 |

**Table S5.3** Power budget, mean B_1_^+^ and SARs for Thelonius model shifted within the birdcage coil.

|  | | **DIZZY** | | | | | | | |
| --- | --- | --- | --- | --- | --- | --- | --- | --- | --- |
| **Relative position** | | **x-25** | **x+25** | **y-25** | **y+25** | **z-50** | **z-25** | **z+25** | **z+50** |
| **Power budget (%)** | Absorbed | 80.7 | 80.2 | 81.1 | 79.0 | 65.1 | 74.0 | 82.3 | 82.2 |
|  | Reflected/Coupled | 3.7 | 3.7 | 3.7 | 5.2 | 18.7 | 9.8 | 2.8 | 4.3 |
|  | Radiated | 10.5 | 11.5 | 10.8 | 11.2 | 12.8 | 12.1 | 10.4 | 10.1 |
|  | Other | 5.1 | 4.6 | 5.3 | 4.6 | 3.4 | 4.1 | 4.5 | 3.4 |
| **Mean B_1_^+^ for 1 W total power (**$\boldsymbol{\mu}$**T/√W)** | | 0.486 | 0.486 | 0.490 | 0.479 | 0.476 | 0.510 | 0.417 | 0.324 |
| **SAR per W total power (W/kg/W)** | Head average | 0.183 | 0.181 | 0.185 | 0.178 | 0.138 | 0.166 | 0.180 | 0.167 |
|  | psSAR_10g_ | 0.503 | 0.470 | 0.484 | 0.517 | 0.674 | 0.622 | 0.511 | 0.616 |
|  | Body average | 0.031 | 0.031 | 0.031 | 0.030 | 0.025 | 0.028 | 0.031 | 0.031 |
| **SAR per mean B_1_^+^ (W/kg/**$\boldsymbol{\mu}$**T^2^)** | Head average | 0.775 | 0.766 | 0.771 | 0.776 | 0.609 | 0.638 | 1.035 | 1.591 |
|  | psSAR_10g_ | 2.130 | 1.990 | 2.016 | 2.253 | 2.975 | 2.391 | 2.939 | 5.868 |
|  | Body average | 0.131 | 0.131 | 0.129 | 0.131 | 0.110 | 0.108 | 0.178 | 0.295 |
| **Ratio psSAR_10g_/ hdSAR** | | 2.75 | 2.60 | 2.62 | 2.90 | 4.88 | 3.75 | 2.84 | 3.69 |

**Table S5.4** Power budget, mean B_1_^+^ and SARs for Dizzy model shifted within the birdcage coil.

|  | | **EARTHA** | | | | | | | |
| --- | --- | --- | --- | --- | --- | --- | --- | --- | --- |
| **Relative position** | | **x-25** | **x+25** | **y-25** | **y+25** | **z-50** | **z-25** | **z+25** | **z+50** |
| **Power budget (%)** | Absorbed | 83.2 | 82.7 | 83.3 | 81.1 | 66.9 | 76.4 | 84.5 | 82.3 |
|  | Reflected/Coupled | 3.6 | 3.2 | 3.7 | 5.1 | 16.7 | 8.7 | 2.9 | 3.9 |
|  | Radiated | 9.4 | 11.0 | 9.8 | 10.4 | 11.9 | 11.3 | 9.5 | 9.2 |
|  | Other | 3.8 | 3.1 | 3.2 | 3.4 | 4.5 | 3.6 | 3.1 | 4.6 |
| **Mean B_1_^+^ for 1 W total power (**$\boldsymbol{\mu}$**T/√W)** | | 0.500 | 0.498 | 0.518 | 0.488 | 0.505 | 0.533 | 0.419 | 0.309 |
| **SAR per W total power (W/kg/W)** | Head average | 0.174 | 0.173 | 0.175 | 0.169 | 0.132 | 0.158 | 0.169 | 0.147 |
|  | psSAR_10g_ | 0.502 | 0.455 | 0.480 | 0.461 | 0.645 | 0.598 | 0.498 | 0.564 |
|  | Body average | 0.027 | 0.027 | 0.028 | 0.027 | 0.022 | 0.025 | 0.028 | 0.027 |
| **SAR per mean B_1_^+^ (W/kg/**$\boldsymbol{\mu}$**T^2^)** | Head average | 0.696 | 0.698 | 0.652 | 0.710 | 0.518 | 0.556 | 0.963 | 1.540 |
|  | psSAR_10g_ | 2.008 | 1.835 | 1.789 | 1.936 | 2.529 | 2.105 | 2.837 | 5.907 |
|  | Body average | 0.108 | 0.109 | 0.104 | 0.113 | 0.086 | 0.088 | 0.159 | 0.283 |
| **Ratio psSAR_10g_/ hdSAR** | | 2.89 | 2.63 | 2.74 | 2.73 | 4.88 | 3.78 | 2.95 | 3.84 |

**Table S5.5** Power budget, mean B_1_^+^ and SARs for Eartha model shifted within the birdcage coil.

|  | | **BILLIE** | | | | | | | |
| --- | --- | --- | --- | --- | --- | --- | --- | --- | --- |
| **Relative position** | | **x-25** | **x+25** | **y-25** | **y+25** | **z-50** | **z-25** | **z+25** | **z+50** |
| **Power budget (%)** | Absorbed | 82.1 | 81.6 | 82.5 | 79.9 | 64.4 | 74.6 | 84.1 | 84.0 |
|  | Reflected/Coupled | 4.1 | 4.5 | 4.2 | 6.1 | 18.8 | 10.3 | 2.9 | 2.5 |
|  | Radiated | 10.1 | 10.8 | 10.0 | 10.7 | 12.1 | 11.5 | 9.8 | 9.4 |
|  | Other | 3.7 | 3.1 | 3.3 | 3.3 | 4.7 | 3.6 | 3.2 | 4.1 |
| **Mean B_1_^+^ for 1 W total power (**$\boldsymbol{\mu}$**T/√W)** | | 0.508 | 0.504 | 0.511 | 0.494 | 0.512 | 0.535 | 0.433 | 0.327 |
| **SAR per W total power (W/kg/W)** | Head average | 0.183 | 0.180 | 0.187 | 0.174 | 0.131 | 0.162 | 0.182 | 0.169 |
|  | psSAR_10g_ | 0.496 | 0.483 | 0.509 | 0.464 | 0.595 | 0.577 | 0.596 | 0.666 |
|  | Body average | 0.023 | 0.023 | 0.023 | 0.023 | 0.018 | 0.022 | 0.024 | 0.024 |
| **SAR per mean B_1_^+^ (W/kg/**$\boldsymbol{\mu}$**T^2^)** | Head average | 0.709 | 0.709 | 0.716 | 0.713 | 0.500 | 0.566 | 0.971 | 1.580 |
|  | psSAR_10g_ | 1.922 | 1.901 | 1.949 | 1.901 | 2.270 | 2.016 | 3.179 | 6.228 |
|  | Body average | 0.089 | 0.091 | 0.088 | 0.094 | 0.069 | 0.077 | 0.128 | 0.224 |
| **Ratio psSAR_10g_/ hdSAR** | | 2.7 | 2.7 | 2.7 | 2.7 | 4.5 | 3.6 | 3.3 | 3.9 |

**Table S5.5** Power budget, mean B_1_^+^ and SARs for Billie model shifted within the birdcage coil.

|  | | **LOUIS** | | | | | | | |
| --- | --- | --- | --- | --- | --- | --- | --- | --- | --- |
| **Relative position** | | **x-25** | **x+25** | **y-25** | **y+25** | **z-50** | **z-25** | **z+25** | **z+50** |
| **Power budget (%)** | Absorbed | 84.4 | 84.3 | 85.4 | 82.6 | 65.1 | 76.7 | 86.6 | 87.0 |
|  | Reflected/Coupled | 3.8 | 3.2 | 2.7 | 4.3 | 13.7 | 7.5 | 2.3 | 2.5 |
|  | Radiated | 7.3 | 8.6 | 8.1 | 7.9 | 9.6 | 9.0 | 7.4 | 6.9 |
|  | Other | 4.5 | 3.9 | 3.8 | 5.2 | 11.6 | 6.8 | 3.7 | 3.6 |
| **Mean B_1_^+^ for 1 W total power (**$\boldsymbol{\mu}$**T/√W)** | | 0.501 | 0.493 | 0.513 | 0.476 | 0.480 | 0.518 | 0.437 | 0.339 |
| **SAR per W total power (W/kg/W)** | Head average | 0.169 | 0.164 | 0.171 | 0.161 | 0.117 | 0.147 | 0.169 | 0.163 |
|  | psSAR_10g_ | 0.525 | 0.573 | 0.543 | 0.641 | 0.615 | 0.625 | 0.471 | 0.489 |
|  | Body average | 0.017 | 0.017 | 0.017 | 0.017 | 0.013 | 0.015 | 0.017 | 0.017 |
| **SAR per mean B_1_^+^ (W/kg/**$\boldsymbol{\mu}$**T^2^)** | Head average | 0.673 | 0.675 | 0.650 | 0.711 | 0.508 | 0.548 | 0.885 | 1.418 |
|  | psSAR_10g_ | 2.092 | 2.358 | 2.063 | 2.829 | 2.669 | 2.329 | 2.466 | 4.255 |
|  | Body average | 0.068 | 0.070 | 0.065 | 0.075 | 0.056 | 0.056 | 0.089 | 0.148 |
| **Ratio psSAR_10g_/ hdSAR** | | 3.1 | 3.5 | 3.2 | 4.0 | 5.3 | 5.3 | 4.3 | 2.8 |

**Table S5.7** Power budget, mean B_1_^+^ and SARs for Louis model shifted within the birdcage coil.

|  | | **DUKE** | | | | | | | |
| --- | --- | --- | --- | --- | --- | --- | --- | --- | --- |
| **Relative position** | | **x-25** | **x+25** | **y-25** | **y+25** | **z-50** | **z-25** | **z+25** | **z+50** |
| **Power budget (%)** | Absorbed | 88.7 | 88.0 | 89.0 | 87.2 | 76.6 | 84.5 | 89.1 | 89.7 |
|  | Reflected/Coupled | 0.9 | 0.9 | 0.9 | 2.3 | 10.2 | 3.8 | 1.2 | 1.8 |
|  | Radiated | 6.3 | 7.6 | 6.6 | 7.2 | 9.5 | 8.4 | 6.0 | 5.6 |
|  | Other | 5 | 3.5 | 3.5 | 3.3 | 3.7 | 3.3 | 3.7 | 2.9 |
| **Mean B_1_^+^ for 1 W total power (**$\boldsymbol{\mu}$**T/√W)** | | 0.468 | 0.460 | 0.470 | 0.419 | 0.435 | 0.475 | 0.421 | 0.345 |
| **SAR per W total power (W/kg/W)** | Head average | 0.152 | 0.145 | 0.151 | 0.145 | 0.118 | 0.138 | 0.149 | 0.144 |
|  | psSAR_10g_ | 0.465 | 0.443 | 0.493 | 0.461 | 0.592 | 0.542 | 0.437 | 0.466 |
|  | Body average | 0.013 | 0.013 | 0.013 | 0.012 | 0.011 | 0.012 | 0.013 | 0.013 |
| **SAR per mean B_1_^+^ (W/kg/**$\boldsymbol{\mu}$**T^2^)** | Head average | 0.694 | 0.685 | 0.684 | 0.826 | 0.624 | 0.612 | 0.841 | 1.210 |
|  | psSAR_10g_ | 2.123 | 2.094 | 2.232 | 2.626 | 3.129 | 2.402 | 2.466 | 3.915 |
|  | Body average | 0.059 | 0.061 | 0.059 | 0.068 | 0.058 | 0.053 | 0.073 | 0.109 |
| **Ratio psSAR_10g_/ hdSAR** | | 3.06 | 3.06 | 3.26 | 3.18 | 5.02 | 3.93 | 2.93 | 3.24 |

**Table S5.8** Power budget, mean B_1_^+^ and SARs for Duke model shifted within the birdcage coil.
